# Life-sustaining therapy limitation in critical illness: unequal weights of demographics, disease severity, and comorbidities

**DOI:** 10.62675/2965-2774.20260348

**Published:** 2026-09-15

**Authors:** Gustavo Luís Hubert Ribeiro, João Gabriel Rosa Ramos, Edison Moraes Rodrigues, Cassiano Teixeira

**Affiliations:** 1 Universidade Federal do Rio Grande do Sul Hospital de Clínicas de Porto Alegre Department of Critical Care Medicine Porto Alegre RS Brazil Department of Critical Care Medicine, Hospital de Clínicas de Porto Alegre, Universidade Federal do Rio Grande do Sul - Porto Alegre (RS), Brazil.; 2 Department of Rehabilitation and Palliative Care, Clínica Florence Salvador BA Brazil Department of Rehabilitation and Palliative Care, Clínica Florence - Salvador (BA), Brazil.

**Keywords:** Critical care, Ethics, Life support care, Withholding treatment, Frailty, Frail elderly

## Abstract

**Objective:**

To identify independent clinical predictors of life-sustaining treatment limitation in critically ill adults and to quantify their relative contribution across predefined conceptual domains.

**Methods:**

We conducted a retrospective cohort study including all adults admitted to a tertiary medical-surgical intensive care unit in Brazil between May 2016 and May 2020. Patients with documented life-sustaining treatment limitations (withholding or withdrawal) were compared with those who received full treatment. Multivariable logistic regression was used to identify independent predictors of life-sustaining treatment limitation, and predictors were grouped into conceptual domains to assess their relative contributions.

**Results:**

Among 6,725 intensive care unit admissions, 336 patients (5.0%) had documented life-sustaining treatment limitation. Compared with patients without limitation, those with life-sustaining treatment decisions were older (median 77 *versus* 67 years; p < 0.001), more frequently frail (Clinical Frailty Scale ≥ 5: 7.6% *versus* 3.1%; p = 0.002), and had more metastatic cancer (15.6% *versus* 5.8%; p < 0.001). Independent predictors of life-sustaining treatment limitation included age ≥ 80 years (OR 2.08; 95%CI 1.32 - 3.28), frailty (OR 2.37; 95%CI 1.53 - 3.71), metastatic cancer (OR 2.38; 95%CI 1.53 - 3.71), medical admission (OR 1.86; 95%CI 1.02 - 3.40), higher SAPS 3 scores (OR 1.02 per point; 95%CI 1.01 - 1.03), and prolonged mechanical ventilation (OR 5.78 for ≥10 days). The presence of any organ support was inversely associated with life-sustaining treatment limitation (OR 0.69; 95%CI 0.49 - 0.97). When predictors were aggregated into conceptual domains and evaluated using Wald χ² proportions, acute illness severity and life-suppor-related variables accounted for approximately 45% of the model signal, followed by demographic factors (≈38%) and chronic comorbidities (≈17%).

**Conclusion:**

Life-sustaining treatment limitation decisions in critically ill adults are influenced by frailty, comorbidities, and acute severity. Grouping predictors into conceptual domains enhances interpretability and may support more structured and transparent decision-making frameworks in intensive care unit settings.

## INTRODUCTION

Life expectancy has been steadily rising in many countries, leading to an increased population of older and multimorbid patients.^(1)^ As a result, intensive care units (ICUs) around the world have had to expand their capacity and rethink clinical policies to keep up with this mounting demand. Estimates suggest that the need for ICU beds may rise by as much as 50% in the coming years.^(2)^ Alongside the increase in admissions, the number of ICU survivors living with significant long-term impairments - such as chronic pain, cognitive decline, and poor quality of life (QoL) - has also gone up. These post-ICU burdens often prompt discussions about the appropriateness of continuing aggressive care, ultimately pushing clinicians to consider limiting life-sustaining treatments (LSTs).

Despite the progress in medical technologies and critical care interventions, ICU teams frequently face uncertainty about the effectiveness and appropriateness of ongoing life support, particularly in patients with dismal prognosis, severe physiological derangement, or poor response to treatment. To navigate this uncertainty, some clinicians rely on time-limited treatment trials (TLTTs), where LSTs are initiated but reassessed after a defined period. However, these strategies often bring up difficult questions: when should life support be scaled back, and under what circumstances should it be withdrawn altogether? Variation in ICU admission criteria, healthcare system structures,^(3–5)^ and the frequent lack of advance directives or documented patient preferences contribute to major differences in how end-of-life (EOL) care is delivered across institutions.^(6)^ Importantly, decisions to limit life support often do not stem solely from clinical factors or patient wishes.^(7,8)^ Instead, they tend to reflect institutional norms, regional practices, and individual physician attitudes toward dying, autonomy, and medical futility. Intensive care units physicians are therefore increasingly tasked with making ethically complex decisions about withholding or withdrawing life support, especially when patients show little or no improvement despite maximal care. In such cases, the focus may shift from extending survival to preventing unnecessary suffering. Yet, these decisions are shaped by a host of uncertainties - ethical, cultural, religious, and prognostic - which makes it hard to lay down clear, universal guidelines.^(8)^

So, the objective of this study was to identify clinical factors associated with decisions to limit life-sustaining treatment in critically ill adults and to explore how these factors cluster into broader conceptual domains relevant to EOL decision-making in the ICU.

## METHODS

### Study design and population

This was a retrospective observational study based on data collected from a single medical-surgical ICU over four years, from May 2016 to May 2020. All adult patients admitted to the ICU during this period were considered eligible for inclusion. The study was approved by the local institutional ethics committee, which granted a waiver of informed consent due to the observational nature of the study. No formal sample size calculation was carried out, as this study aimed to explore associations within an existing database rather than to test a predefined hypothesis.

### Data collection

A standardized set of clinical and demographic variables was extracted from the ICU database. The following patient characteristics were recorded: age, gender, reason for ICU admission - categorized as elective surgery, emergency surgery, sepsis, neurological causes, gastrointestinal or liver dysfunction, hematological disease, renal or metabolic dysfunction, respiratory failure, oncological disease, obstetric causes, or trauma - length of hospital stay prior to ICU admission, and baseline frailty using the Clinical Frailty Scale (CFS) - categorized as non-frail (CFS 0 - 3), pre-frail (CFS 4), and frail (CFS ≥ 5). The Sequential Organ Failure Assessment (SOFA) score was calculated within the first 24 hours after ICU admission to estimate disease severity. Additionally, the Simplified Acute Physiology Score III (SAPS III) was collected when available.

We also documented the use of major life-sustaining therapies, including any duration of non-invasive or invasive mechanical ventilation (MV) (via endotracheal tube or tracheostomy), vasoactive drug infusion, and renal replacement therapy (RRT). For each intervention, both the ICU day of initiation and total duration (in hours) were recorded and later converted into days. Intensive care units outcomes (survival or death) were registered for all patients.

Limitation of life-sustaining therapy - defined as withholding or withdrawal of ongoing or planned life-sustaining therapies - was documented only if explicitly recorded in the patient chart, in line with international guidelines, and previous studies.^(3)^ Decisions regarding limitation of life-sustaining treatment were prospectively entered into the ICU electronic database as part of routine clinical documentation. They were subsequently audited by designated ICU physicians responsible for data quality assurance, ensuring consistency and reliability of the recorded information.

### Statistical analysis

Patients were categorized based on whether LST limitation – defined as withholding or withdrawal – was implemented during their ICU stay. Baseline characteristics, clinical variables, and outcomes were compared between groups using descriptive statistics. Categorical variables were expressed as counts and percentages and compared using the chi-squared test. Continuous variables were reported as medians with interquartile ranges (IQRs) and compared using the Mann-Whitney U test.

To identify independent predictors of LST limitation, a multivariable logistic regression model was constructed. Variables were selected a priori based on clinical judgment and significance in univariate analysis. They included age, gender, frailty (CFS), comorbidities, ICU admission type, SOFA and SAPS 3 scores, MV, vasoactive drug use, and the need for organ support. To avoid redundancy and collinearity among predictors, composite indices summarizing comorbidity burden, such as the Charlson Comorbidity Index, were excluded from the final multivariable model when their individual components were included as separate covariates. This strategy aimed to improve model parsimony and enhance clinical interpretability of the estimated associations. The presence of at least one life-support modality - defined as the use of MV, vasoactive agents, or RRT - was modeled as a binary variable. Odds ratios (ORs) with 95% confidence intervals (95%CIs) were reported. No imputation was used for missing data.

Although the Charlson Comorbidity Index was initially used to describe overall comorbidity burden in the univariate analysis, it was not included in the final multivariable model due to potential collinearity with individual comorbid conditions. Instead, metastatic cancer was retained as a representative variable within the comorbidity domain, given its strong and independent association with life-sustaining treatment limitations.

To enhance interpretability, predictors were then classified into three conceptual domains: (1) demographic factors (age, frailty), (2) acute illness severity and support requirements (SAPS 3, admission type, MV duration, need for organ support), and (3) comorbidities (metastatic cancer). For domain-level interpretation, predictors were grouped a priori into conceptual categories, and the relative contribution of each domain was quantified as the sum of Wald χ² statistics divided by the total model χ². Multicollinearity was checked before final modeling, and interaction terms were not tested.

All analyses were conducted using R software version 3.5.1 (R Foundation for Statistical Computing, Vienna, Austria).^(9)^ A two-sided p-value < 0.05 was considered statistically significant.

## RESULTS

A total of 6,725 ICU admissions were included, of which 336 (5.0%) involved life-sustaining treatment limitation. Patients in the LST limitation group were older, more frequently frail, and had a higher burden of comorbidities compared with those receiving full treatment. Baseline characteristics and univariate comparisons are presented in table 1, while the results of the multivariable logistic regression analysis are shown in table 2.

**Table 1:** Baseline characteristics and univariate analysis of predictors of life-sustaining treatment limitation in intensive care unit patients

Variable		LST limitation (n = 336)	Full treatment (n = 6,389)	p value
Female gender		179 (48.9)	2,968 (46.5)	0.36
Age		77 [65 - 84]	67 [52- 78]	< 0.001
	≤ 59	112 (30.6)	2,865 (44.8)	–
	60 - 79	194 (53.0)	3,026 (47.3)	–
	≥ 80	59 (16.1)	494 (7.7)	–
Clinical Frailty Scale		3 [2 - 6]	2 [1 - 4]	< 0.001
	Non-frail (CFS 1 - 3)	316 (86.3)	5,929 (92.8)	–
	Pre-frail (CFS 4)	22 (6.0)	258 (4.0)	–
	Frail (CFS ≥ 5)	28 (7.6)	195 (3.1)	–
Charlson Index		2 [1 - 4]	1 [0 - 3]	< 0.001
	Charlson = 0	46 (12.5)	1,404 (21.9)	–
	Charlson = 1	43 (11.7)	875 (13.6)	0.06
	Charlson ≥ 2	277 (75.6)	4,155 (65.0)	0.09
Comorbidities				
	AIDS	22 (6.0)	377 (5.5)	0.90
	Congestive heart failure	51 (13.9)	706 (10.3)	0.16
	COPD Gold C/D	32 (8.7)	514 (7.5)	0.81
	End-stage renal disease	18 (4.9)	598 (8.7)	< 0.001
	Dementia	16 (4.3)	85 (1.3)	< 0.001
	Local cancer	50 (13.6)	799 (11.7)	0.73
	Metastatic cancer	57 (15.6)	373 (5.8)	< 0.001
	Hematologic malignancy	28 (7.6)	459 (6.7)	0.91
	Cirrhosis	28 (7.6)	471 (6.8)	0.97
Hospital LOS before ICU (days)		–	–	0.48
ICU readmission		43 (11.7)	682 (9.9)	0.51
Type of admission				< 0.001
	Emergency surgery	13 (3.5)	465 (6.8)	–
	Elective surgery	5 (1.4)	392 (5.7)	0.98
	Medical	318 (95.1)	5526 (80.8)	0.04
SAPS 3		68.5	66.2	0.002
SOFA at 24 hours		7.9	7.4	0.25
Organ support use				< 0.001
	No organ support	35 (9.5)	1,635 (25.5)	–
	≥ 1 support modality	328 (89.6)	4,669 (73.0)	0.03
	Vasoactive drugs	213 (58.2)	2,946 (43.1)	< 0.001
Mechanical ventilation duration				
	< 1 day	14 (3.8)	484 (7.5)	–
	1 - 3 days	82 (22.4)	1,553 (24.3)	0.03
	4 - 9 days	102 (27.8)	1,103 (17.2)	< 0.001
	≥ 10 days	114 (31.1)	839 (13.1)	< 0.001
Renal replacement therapy		80 (21.8)	1,509 (23.6)	0.40
Tracheostomy		19 (5.2)	180 (2.8)	< 0.001

LT - life-sustaining treatment; IQR – interval interquartile; CFS - Clinical Frailty Scale; COPD - chronic obstructive pulmonary disease; LOS - length of stay; ICU - intensive care unit; SAPS - Simplified Acute Physiology Score; SOFA - Sequential Organ Failure Assessment. Results expressed as n (%), median [interquartile range] or median.

**Table 2:** Multivariable logistic regression analysis of predictors of life-sustaining treatment limitation

Variable		Adjusted OR (95%CI)	p value
Age			
	60 - 79 years	1.36 (1.03 - 1.80)	0.02
	≥ 80 years	2.08 (1.32 - 3.28)	0.002
Frailty (CFS ≥ 5)		2.37 (1.53 - 3.71)	0.002
Metastatic cancer		2.38 (1.53 - 3.71)	< 0.001
Medical admission		1.86 (1.02 - 3.40)	0.04
SAPS 3 (per point)		1.02 (1.01 - 1.03)	0.002
Organ support (≥ 1 modality)		0.69 (0.49 - 0.97)	0.03
Mechanical ventilation duration			
	1 - 3 days	1.88 (1.05 - 3.37)	0.03
	4 - 9 days	3.08 (1.69 - 5.61)	< 0.001
	≥ 10 days	5.78 (3.14 - 10.63)	< 0.001
Tracheostomy		0.94 (0.54 - 1.64)	0.83

OR - odds ratio; 95%CI - 95% confidence interval; CFS - Clinical Frailty Scale; SAPS - Simplified Acute Physiology Score.

In univariate analysis, LST limitation was significantly associated with age ≥ 80 years (16.1% *versus* 7.7%; p < 0.001), frailty (CFS ≥ 5 in 7.6% *versus* 3.1%; p = 0.002), and metastatic cancer (15.6% *versus* 5.8%; p < 0.001). Patients requiring prolonged MV (≥ 10 days) were disproportionately represented in the LST limitation group (31.1% *versus* 13.1%; p < 0.001). Conversely, those with no organ support were less likely to have limitations documented (9.5% *versus* 25.5%; p = 0.03).

In the multivariate adjusted analysis, LST limitation was most strongly associated with prolonged MV, with a stepwise increase in odds from 1.88 (95%CI 1.05 - 3.37) for 1 - 3 days to 5.78 (95%CI 3.14 - 10.63) for ≥ 10 days, followed by markers of baseline vulnerability such as frailty (OR 2.37; 95%CI 1.53 - 3.71) and age ≥ 80 years (OR 2.08; 95%CI 1.32 - 3.28), while medical admission (OR 1.86; 95%CI 1.02 - 3.40) and higher acute physiological severity (SAPS 3; OR 1.02 per point; 95%CI 1.01 - 1.03) showed more modest but independent associations.

When predictors were grouped into predefined conceptual domains and their relative contributions were assessed using Wald χ² statistics, variables related to acute illness severity and life support accounted for the largest share of the model signal (approximately 45%), followed by demographic factors, including age and frailty (approximately 38%), and chronic comorbidities, primarily metastatic cancer (approximately 17%) (Figure 1).

**Figure 1:**
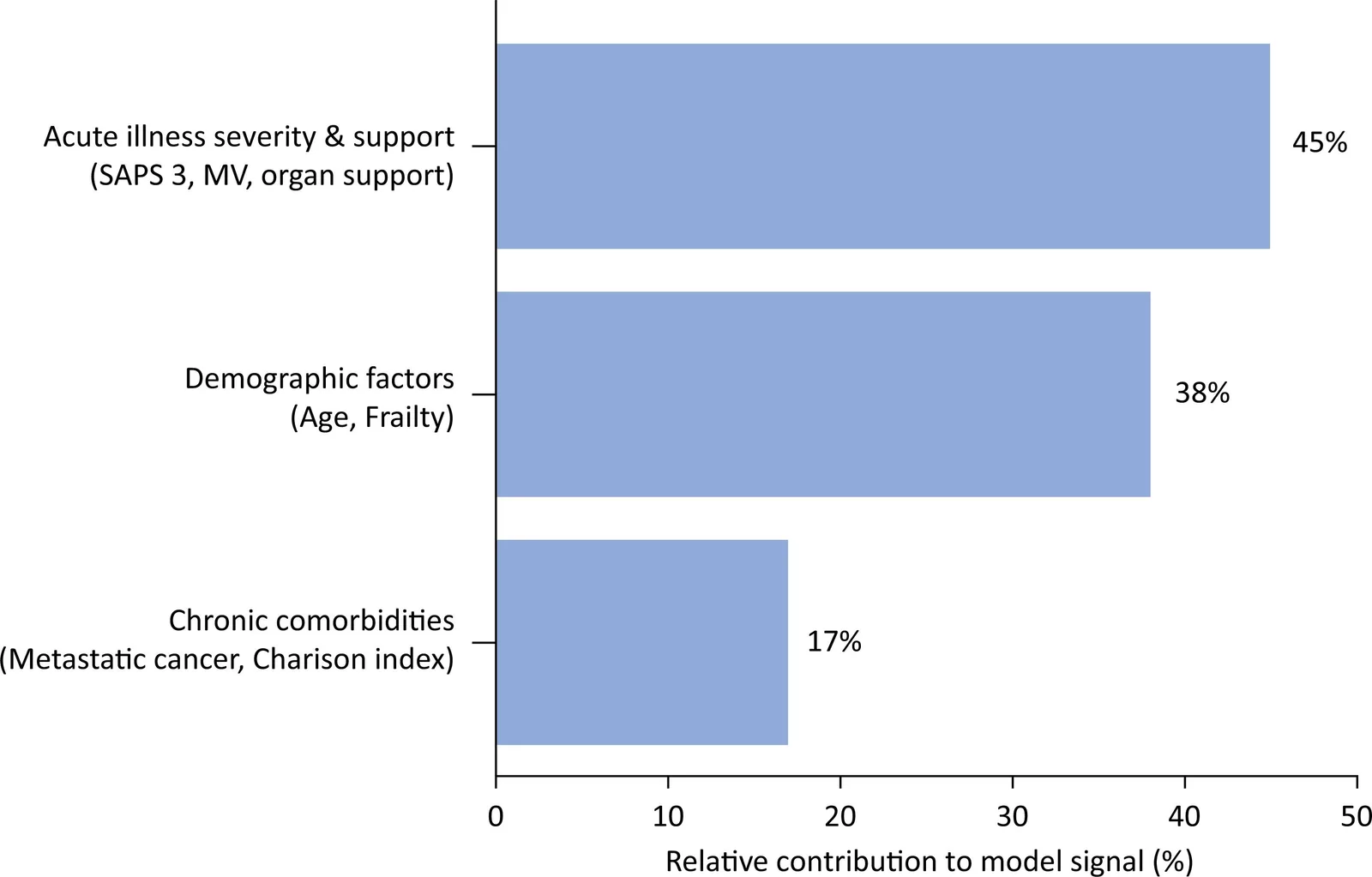
Relative contribution of conceptual predictor domains to life-sustaining treatment limitation from the multivariable logistic regression model

SAPS - Simplified Acute Physiology Score; MV - mechanical ventilation.

The figure displays the relative contribution of predefined predictor domains to the multivariable logistic regression model, calculated as the sum of Wald Chi-squared (χ^2^) statistics for each domain divided by the total model χ^2^. This representation is intended for descriptive comparison of model contributions only and does not imply explained variance or causality.

## DISCUSSION

This study identified several clinical factors independently associated with decisions to limit LSTs in critically ill patients. Among them, age ≥ 80 years, high frailty scores, and the presence of metastatic cancer stood out as the strongest predictors. In addition, indicators of acute physiological stress - such as elevated SAPS 3 scores and prolonged MV - were also associated with a higher likelihood of LST limitation. Interestingly, the need for any organ support early in the ICU stay was inversely associated with limitation, possibly reflecting a tendency to initiate full treatment before reevaluation. Grouping these predictors into conceptual domains highlighted the interplay between baseline vulnerability, illness severity, and long-term prognosis in shaping EOL decisions.

These findings are consistent with prior research demonstrating that frailty and malignancy strongly influence limitation of therapy in the ICU.^(10–12)^ Previous European studies, such as ETHICUS and ETHICUS-2, showed considerable variation in limitation practices across countries and institutions, often driven by differences in policy and local culture rather than patient condition alone.^(4,13,14)^ Although age is often cited as a factor in decision-making, current guidelines emphasize that it should not be used in isolation.^(15–17)^ The idea of an "ICU trial" - initiating full support followed by reassessment within the first days - has been recommended particularly for patients with limited prognosis, including those with advanced cancers.^(18,19)^ Our findings align with this concept, suggesting that decisions to withhold or withdraw treatment are often made after a period of intensive support and reassessment.

Mechanistically, the convergence of multiple stressors - chronological age, functional decline, and acute physiological derangement - is likely to influence decision-making regarding limitation of therapy.^(20)^ Frailty is generally considered a marker of diminished physiological reserve and correlates with poor outcomes, even in patients who survive the ICU.^(21,22)^ Similarly, metastatic cancer is associated with not only a shortened life expectancy but also therapeutic limitations unrelated to acute illness. The strong association between MV duration and LST limitation in our study may represent a plausible explanatory interpretation related to the perception of therapeutic futility, particularly when prolonged ventilatory support does not result in clinical improvement. Conversely, the inverse association between early organ support initiation and LST limitation may represent a plausible explanatory hypothesis, reflecting an initial commitment to curative or life-prolonging intent at ICU admission, with limitation or withdrawal decisions potentially emerging later in the clinical course as response to therapy and prognosis become clearer; however, this temporal relationship cannot be established in the present study. Importantly, this finding should be interpreted considering the temporal structure of ICU decision-making and the partial collinearity between early organ support initiation and downstream markers of treatment intensity (such as duration of MV or need for prolonged support), which may influence the direction and magnitude of observed associations in multivariable models.^(23,24)^ In addition, the observed rate of 5% is lower than that usually reported in American and European cohorts (10 - 20%),^(25,26)^ which may reflect underreporting. However, cultural values, institutional practices, and healthcare system incentives could also have influenced these findings.^(27)^

Studies led by Brazilian investigators have highlighted that decisions to withhold or withdraw life-sustaining treatment in the ICU are relatively frequent and closely linked to disease severity, organ dysfunction, and underlying comorbidities. In a large multinational cohort coordinated by Brazilian authors, life-sustaining treatment limitation occurred in approximately 13% of ICU admissions. It was present in 40% of non-survivors, with hospital mortality reaching 69% among patients with such decisions.^(28)^ Complementary Brazilian data further show that withdrawal of MV represented about 11% of patients with treatment limitations and was typically performed after sustained therapeutic trials, with no increase in hospital mortality after propensity score matching, reinforcing the concept that treatment limitation decisions often emerge later in the ICU course and are embedded within a structured, reassessment-based palliative approach.^(29)^

This study has several strengths. It included a large, unselected cohort from a mixed medical-surgical ICU, capturing real-world decision-making. Data were extracted from structured documentation aligned with international guidelines, ensuring reliability of LST records. Moreover, our analytic strategy combined statistical significance with clinical interpretation, grouping predictors into meaningful domains and estimating their relative contributions. However, limitations must be acknowledged. First, the retrospective design precludes assessment of the timing and context in which limitation decisions were made. Second, we did not capture patient or surrogate preferences, which play a crucial role in EOL care, nor did we capture the decision-making process involved in those LST limitation decisions. Third, although frailty was assessed using the CFS, inter-rater variability and retrospective scoring may have affected classification accuracy. Fourth, patients were considered to have undergone LST limitations only when a formal decision was clearly documented in the medical record. However, information on the timing of such decisions relative to ICU admission or initiation of organ support was not available. Fifth, this was a single-center study, and local practice patterns may limit generalizability. Sixth, the inclusion of a composite variable representing any organ support utilization may have introduced partial overlap with individual support-related variables (such as MV duration or vasoactive drug use), and its inverse association with treatment limitation should therefore be interpreted cautiously, as a descriptive marker of early therapeutic intent rather than as an independent or causal effect. Finally, the study outcome may be influenced by time-related bias and competing risks, as patients with greater illness severity may die early during the ICU stay before a formal limitation-of-care decision is documented. As a result, associations involving time-dependent exposures - such as organ support use or its duration - may partially reflect clinical trajectory rather than baseline determinants of decision-making, particularly in the case of the observed low odds ratio among patients receiving at least one form of organ support. These findings should therefore be interpreted with caution, as they may reflect competing-risk mechanisms rather than true protective effects. Although time-to-event analyses using competing-risk models (e.g., Fine-Gray) would be methodologically appealing, the lack of precise information on the timing of limitation-of-care documentation relative to ICU admission and initiation of organ support precluded such analyses in the present study.

## CONCLUSION

Decisions to limit life-sustaining treatment in the intensive care unit are multifactorial, driven by a combination of age, frailty, acute severity, and underlying prognosis. Grouping these predictors into clinical domains may enhance our understanding of the rationale behind treatment limitation and improve communication with patients and families. Future prospective studies are needed to evaluate how structured reassessment, early patient-centered goals of care, and standardized frailty screening might guide more consistent and transparent decision-making.

## Data Availability

The contents underlying the research text are included in the manuscript.
